# SLEEP AND SEXUALITY AMONG CAREGIVERS AND THEIR PARTNERS

**DOI:** 10.1093/geroni/igad104.1715

**Published:** 2023-12-21

**Authors:** Christine Proulx, Hanamori Skoblow, Amy Rauer

**Affiliations:** University of Vermont, Burlington, Vermont, United States; University of Missouri, Columbia, Missouri, United States; University of Tennessee, Knoxville, Tennessee, United States

## Abstract

Older adult caregivers have increased risk of disturbed sleep, which is associated with declines in sexual frequency. Spousal caregivers engage in less sexual activity than caregivers of non-spouses. We examined whether the associations between sleep behaviors (duration, napping frequency, feeling rested) and couples’ sexual frequency, quality, and pleasure were similar among dyads in which one spouse was providing care to a spouse (n = 146) or a parent or other care recipient (n = 211). Using Wave 2 of the NSHAP and actor-partner interdependence models, we found significant differences (p <.01) between dyads. For all spouses, only frequency of sharing a bed was related to frequency of sexual activity in the previous 12 months. Sleep duration was not associated with any outcomes. In both groups, frequency of sharing a bed was positively associated with reports of physical pleasure (b’s ranged from .20 to .50). In spousal caregiver couples, caregivers’ greater napping frequency was negatively associated (b = -.29, p <.01) with their partner’s reports of a physically pleasurable relationship, and their reports of feeling rested were positively (b = .17, p < .01) associated with their own reports of pleasure. For couples in which the caregiver was caring for a non-spouse, there were actor effects for caregivers’ and noncaregivers’ napping frequency on lower reports of sexual relationship quality (b = .19 and .49, respectively), and a partner effect for non-caregivers (b = .25). These results point to the importance of understanding the context of caregiving, sleep behaviors, and sexual relationships.

